# Analysis of visual evoked potentials in patients with neurofibromatosis type 1: new concepts

**DOI:** 10.3389/fneur.2024.1410101

**Published:** 2024-07-22

**Authors:** Jasna Jancic, Nikola Zarkovic, Blazo Nikolic, Nikola Ivancevic, Branislav Rovcanin, Dejan Nesic

**Affiliations:** ^1^Clinic of Neurology and Psychiatry for Children and Youth, Belgrade, Serbia; ^2^Faculty of Medicine, University of Belgrade, Belgrade, Serbia; ^3^Center for Endocrine Surgery, Clinical Center of Serbia, Belgrade, Serbia; ^4^Institute of Medical Physiology, Belgrade, Serbia

**Keywords:** neurofibromatosis type 1, visual evoked potentials, P100 latency, optic pathway gliomas, cognitive disorders

## Abstract

**Introduction:**

Neurofibromatosis type 1 (NF type 1) is an autosomal dominant disease with typical clinical manifestations, such as skin lesions, Lisch nodules, optic pathway gliomas, and neurofibromas, caused by the mutation of the NF1 gene. Visual evoked potentials (VEP) present a measure of the electrophysiological response of visual cortex to a visual stimulus. The role of VEP in the pathophysiology of NF type 1 is very complex and requires additional research.

**The Aim:**

We examined the differences between NF type 1 patients with normal and altered VEP and analyzed the correlation between the prolongation of P100 latency and disease severity.

**Materials and methods:**

Two groups were formed: a control group and a study group with NF type 1 patients. Based on the control group analysis, a threshold value for a normal VEP finding of 116 ms was obtained, and it was used to divide the study group into subgroups with normal and altered VEP. We proceeded with examining the differences in clinical manifestations of the disease between the subgroups, after which we checked if there is a correlation between the prolongation of the P100 latency and the severity of the clinical picture according to the Riccardi scale. Statistical analysis was performed using the Pearson chi-square test and the Spearman correlation test in the program SPSS 28.0, with levels of statistical significance *p* = 0.05 and *p* = 0.001.

**Results:**

In the group with the abnormal VEP we found a statistically significant more frequent occurrence of optic tract glioma (*p* = 0.008), tumors (*p* = 0.032), epilepsy (*p* = 0.043), and cognitive disorders (*p* = 0.028), while the other clinical signs had an equal prevalence in both groups. A moderately strong correlation (*r*_s_ = 0.665) was observed between the prolongation of P100 latency and the severity of the clinical picture.

**Conclusion:**

Our results showed the important role of VEP in the description of clinical phenotypes of NF type 1. The authors of the study propose VEP to be included in the diagnostic algorithms designed for patients with NF type 1.

## 1 Introduction

Neurofibromatosis type 1 (NF type 1) is an autosomal dominant disease characterized by typical clinical manifestations, such as café-au-lait macules, skin fold freckles, Lisch nodules, optic pathway gliomas (OPG) and neurofibromas (1). The estimated prevalence of this disease in the general population is 1 per 3,000 inhabitants, making NF type 1 one of the most common genetic diseases in neurology and human medicine (2). This condition is caused by an inherited or *de novo* mutation of the *NF1* gene located on the long arm of chromosome 17 (3). The product of this gene, neurofibromin, is an essential tumor-suppressor protein that inhibits the activation of the Ras/Raf/MEK/ERK signaling pathway, which is involved in numerous critical cellular processes, especially cell proliferation (4, 5). As a consequence of the mutation, the protein product of the *NF1* gene becomes inactive, resulting in excessive activation of the aforementioned signaling pathway that promotes cell division, which explains the increased incidence of numerous benign and malignant tumors in people with NF type 1 (6). Given the widespread expression of the *NF1* gene in numerous tissues, it is not surprising that this disease is accompanied by a vast range of somatic, cognitive, and behavioral symptoms (7).

Visual evoked potentials (VEP) represent the application of visual stimuli to detect the electrophysiological response of the visual cortex, which is registered with the help of electrodes placed on the scalp (8). There are two primary modalities of visual evoked potentials: (1) flash visual evoked potentials (F-VEP), and (2) pattern-reversal visual evoked potentials (PR-VEP) (9). The latter method, in which a visual stimulus is displayed as a chessboard with alternating black and white fields, is mainly used in clinical practice with cooperative patients due to greater precision and sensitivity (10, 11). After applying a visual stimulus, the information travels along the optic pathway to the visual cortex, where an electrical response is generated in the form of two negative (N75 and N145) and one positive wave (P100) that appear successively after 75 ms, 100 ms, and 145 ms (12). Because of negligible intra- and inter-individual variations, the P100 wave has the greatest diagnostic value while assessing the integrity of the visual pathway (13).

Visual evoked potentials are traditionally utilized in patients with NF type 1 to diagnose and then monitor the progression of OPG (14, 15). However, studies conducted by Taylor (16), North et al. (17), and Dilts et al. (18) showed that pathological VEP can be found in as many as half of the patients suffering from NF type 1, which far exceeds the prevalence of OPG. On the other hand, the study conducted by Castricum et al. (19) showed that altered VEP in patients with NF type 1 can be associated with cognitive deficits and learning disorders. Bearing in mind all of the above, it is without doubt that the role of VEP in the neuropathophysiology of NF type 1 is highly complex and needs further investigation.

Our study aimed to analyze the diagnostic value of VEP in patients suffering from NF type 1, whereby two approaches were defined: (1) examining the differences in clinical manifestations between patients with normal and altered VEP findings, as well as (2) evaluating the correlation between pathological VEP findings and the severity of the clinical picture.

## 2 Materials and methods

Our retrospective study included a total number of 60 patients who were diagnosed with NF type 1 from January 1, 2012, until December 31, 2022. Inclusion criteria were: (1) a diagnosis of NF type 1 made based on the revised criteria of the international consensus group (20), and (2) a carried out VEP analysis. The exclusion criteria from the study were: (1) a diagnosis of another disease with café-au-lait macules as a clinical feature (Legius syndrome), and (2) the existence of ophthalmologic disorders that could affect the validity of the VEP results (amblyopia and reduced visual acuity).

Informed consent was obtained from all patients, and the study was approved by the Ethics Committee of the hospital. Data on the clinical manifestations of NF type 1 were collected for all patients by reviewing the medical documentation available in the Health Information System (medical reports and discharge letters for hospitalized patients). The VEP analysis was conducted in the Clinic’s neurophysiological laboratory using the PR-VEP modality. A CRT monitor was placed at a distance of 100 cm from the examinees, on which a chess board with black and white fields of size 20′, brightness intensity of 50 cd/m^2^, and contrast of 80% was displayed. The fields changed colors (black to white, and white to black) with frequency of 2 Hz. In the center of screen, a red dot was shown, and the examinees were obliged to fixate it throughout the examination. The displayed pattern filled the entirety of the subject’s field of vision, with visual angle values of at least 15°. Two modes of stimulation were used, monocular and binocular, and the entire analysis lasted 300 ms. Four electrodes (Oz, O1, O2, and Fz) were placed on the scalp of the patients following the standards of the 10–20 system. In our research, only the results obtained from the Oz electrode were observed. All technical parameters related to image quality were adjusted according to the recommendations of the International Society for Clinical Electrophysiology of Vision (21).

Before the statistical data processing, we defined the criteria based on which the patients from the examined group were divided into a subgroup with normal and a subgroup with pathological findings of VEP. One of the main characteristics of an abnormal VEP finding is the prolongation of the time until the appearance of the first positive wave, known clinically as P100 latency (22). According to current recommendations, the sum of the mean value and 2.5 standard deviations is the cut-off value for a normal finding of P100 latency (8). For these purposes, a particular control group was formed, which consisted of 50 healthy subjects who underwent VEP analysis at our Clinic. As the normal electrophysiological pattern of VEP is formed from age five and persists until age 60 (11), the criterion for inclusion in the control group was an age greater than five and less than 60 years old. The primary criterion for exclusion was a diagnosis of ophthalmological, neurological, and psychiatric diseases that are known to adversely affect the results of VEP. After having analyzed the data collected from the control group, the mean value for P100 latency (during binocular stimulation) was 107.25 ms with a standard deviation of 3.5 ms. Using the formula, the threshold value for pathologic P100 latency was set at 116 ms.

**Figure 1:**
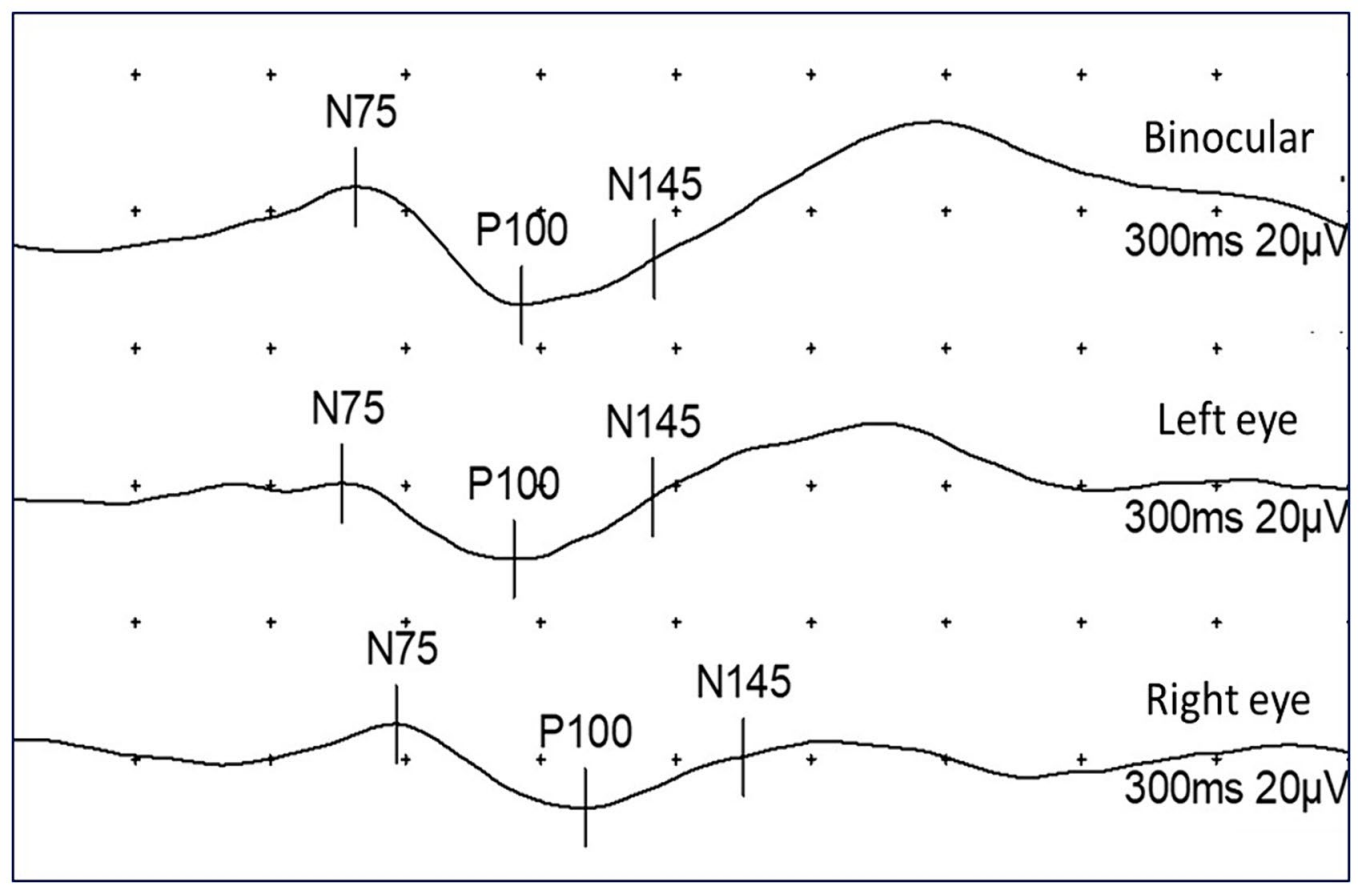
Representation of PR-VEP results using binocular and monocular stimulation. Normal finding in binocular and left-eye stimulation, with pathological prolongation of P100 latency on right eye (P100 = 130 ms).

The first objective of our study was to examine the difference in the frequency of specific clinical characteristics of NF type 1 between the group of patients with physiological and pathological findings of VEP. All patients who, during binocular stimulation, had P100 latency values equal to or greater than 116 ms were classified into the group with pathological VEP findings. Remaining patients were classified into the group with normal VEP (Figure 1). Furthermore, 2 × 2 contingency tables were formed for each clinical feature of interest (Lisch nodules, neurofibromas, OPG, bone lesions, tumors, epilepsy, and cognitive impairment), and the difference in their incidence between the groups with normal and abnormal VEP was analyzed using the Pearson chi-square test of homogeneity.

The second goal of our study included the analysis of a correlation between the prolongation of P100 latency and the severity of the clinical picture in patients with NF type 1. The Riccardi scale with four grades was used to evaluate and grade the clinical picture according to the severity (23). Grade 1 (minimal NF type 1) includes patients with café-au-lait macules and skin fold freckles, while grade 2 (mild NF type 1) implies the existence of neurofibromas without significant somatic and neurological complaints. Grade 3 (moderate NF type 1) includes patients with more severe clinical manifestations (OPG, bone lesions, cognitive impairments) that reduce the patient’s functionality. At the very end, grade 4 (severe NF type 1) consists of patients with serious complications of the disease (pharmacoresistant epilepsy, brain tumors, and malignant tumors) that are difficult to treat and shorten life expectancy. For patients from all four groups, P100 latency values were plotted on the y-axis of the Scatter plot graph, and then the Spearman rank correlation coefficient was calculated.

Statistical data analysis was performed in the computer program SPSS 28.0, with *p* < 0.05 for the Pearson chi-square test (for the level of freedom df = 1) and *p* < 0.001 for the Spearman correlation test taken as statistically significant.

## 3 Results

**Figure 2:**
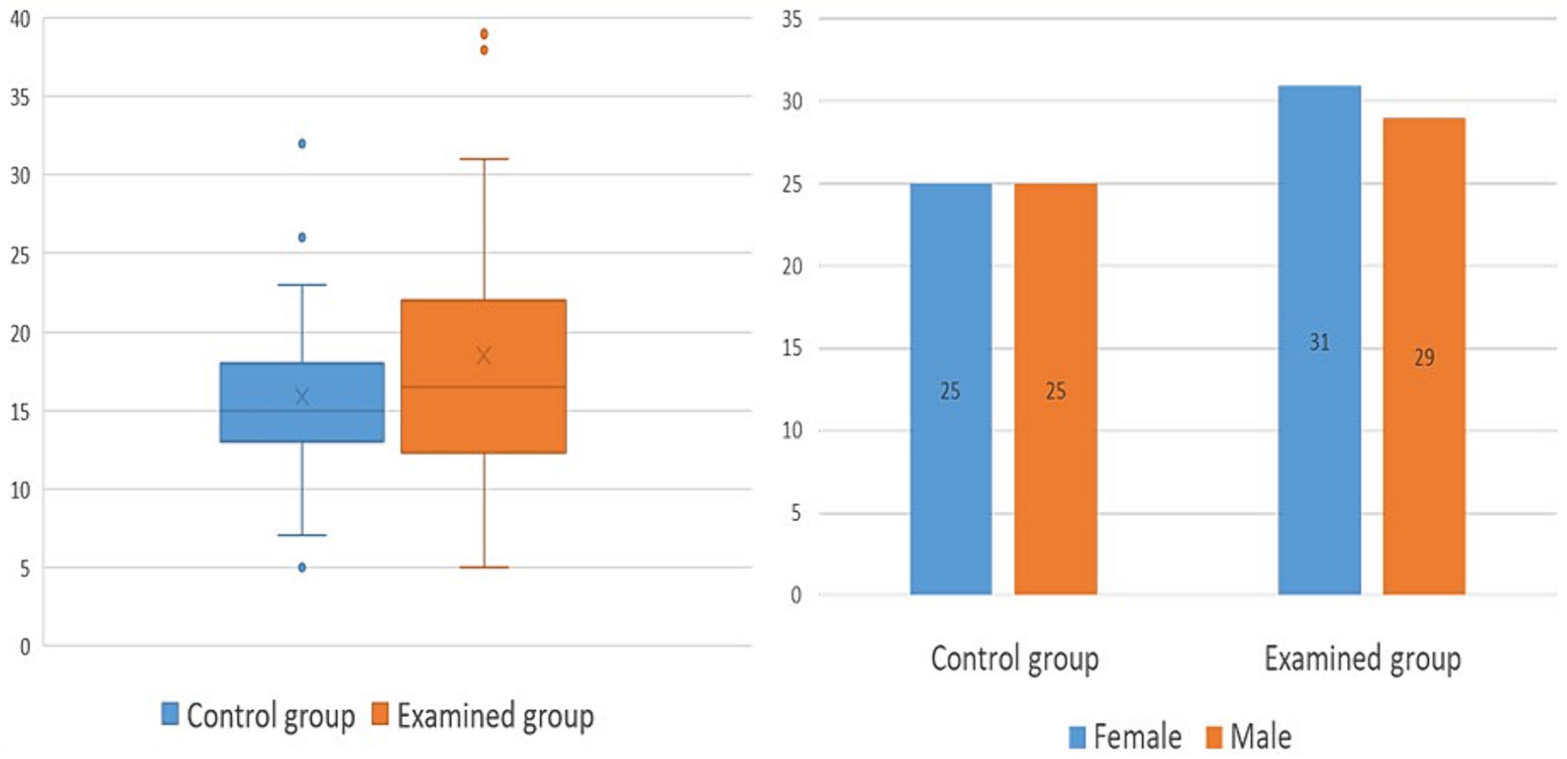
Demographic characteristics of control and examined group; chart 2A demonstrates age, while chart 2B shows gender structure.

The demographic characteristics of the control and test groups were initially analyzed (Figure 2). The average age in the control group was 15.8 years (SD = 7 years), while the average age in the examined group of NF type 1 patients was 18.5 years (SD = 8.5 years). There was no statistically significant difference in age between examined and control groups (*p* = 0.07). For the gender structure, the control group consisted of 25 female (50%) and 25 male (50%) examinees, while the test group included 31 female (52%) and 29 male (48%) patients.

**Figure 3:**
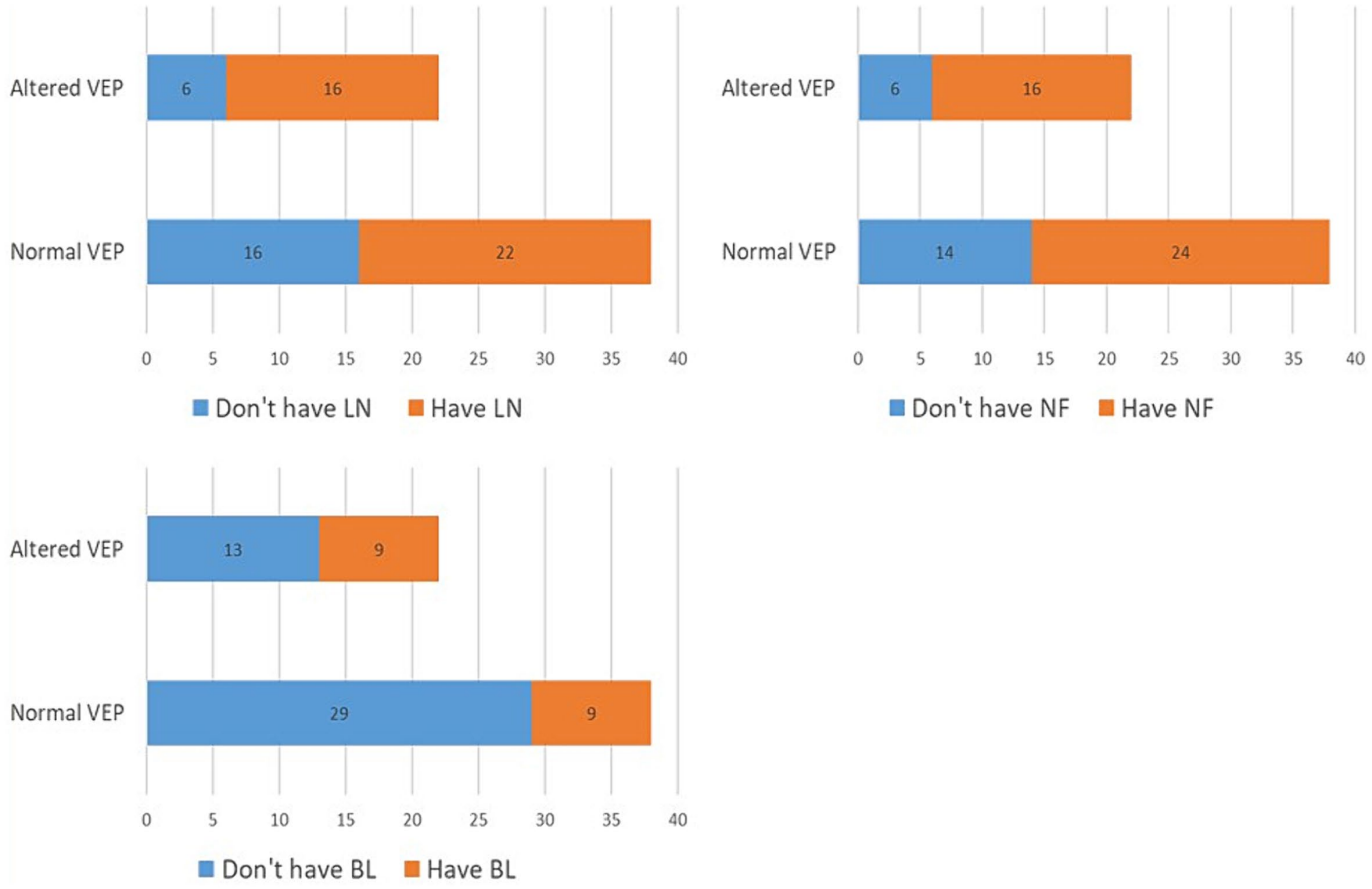
Prevalence of Lisch nodules (2A), neurofibromas (2B) and bone malformations (2C) in groups with normal and abnormal VEP. VEP, visual evoked potentials; LN, Lisch nodules; NF, neurofibromas; BL, bone lesion.

**Figure 4:**
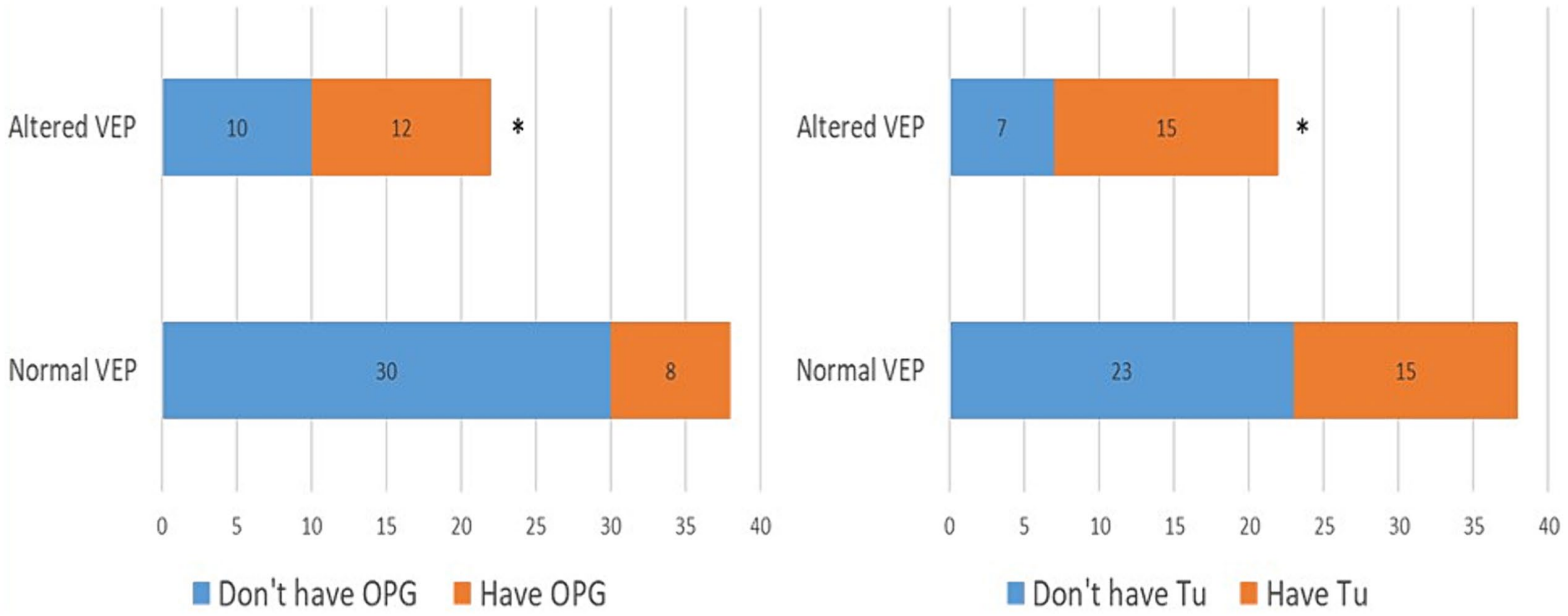
Prevalence of optic pathway gliomas (3A) and other tumors (3B) in groups with normal and abnormal VEP. VEP, visual evoked potentials; GOP, optic pathway gliomas; Tu, tumors. * demonstrates statistically significant more frequent occurrence of optic pathway gliomas (*χ*^2^ = 7.03, *p* = 0.008) and tumors (*χ*^2^ = 4.59, *p* = 0.032) in group with pathologic VEP findings.

**Figure 5:**
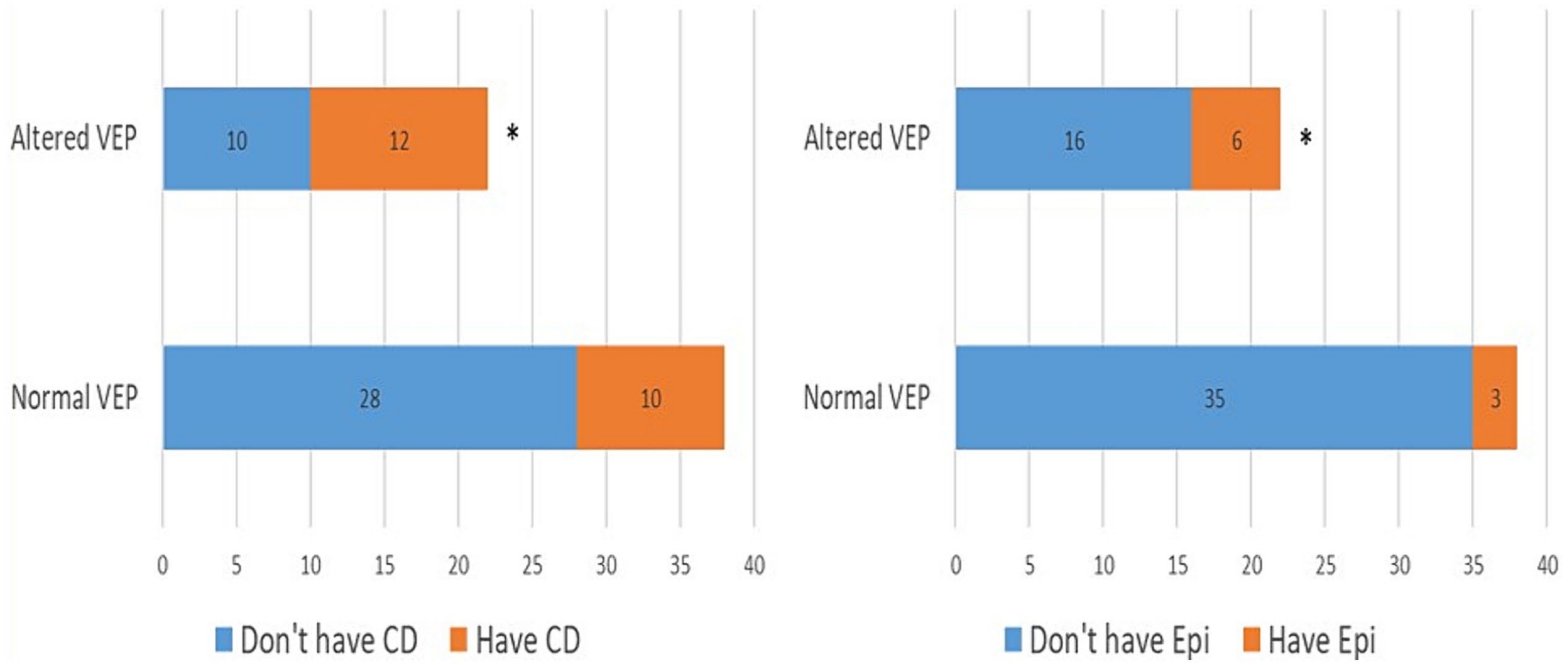
Prevalence of epilepsy (4A) and cognitive disorders (4B) in groups with normal and abnormal VEP. VEP, visual evoked potentials; Epi, epilepsy; CD, cognitive disorder. * demonstrates statistically significant more frequent occurrence of epilepsy (*χ*^2^ = 4.10, *p* = 0.042) and cognitive impairment (*χ*^2^ = 4.78, *p* = 0.028) in group with pathologic VEP findings.

In the further course of the research, we examined (1) the frequency of clinical manifestations of NF type 1, and (2) whether there is a difference in their distribution between the subgroups with physiological and pathological findings of VEP. All patients had café-au-lait macules and skin fold freckles; 67% of patients had neurofibromas, 63% had Lisch nodules, 33% had OPG, 30% had characteristic bone lesions, 50% had other tumors, 15% of patients had epilepsy and finally, as many as 37% of patients had cognitive disorders (Table 1). Using the Chi-square test, the following results were obtained: (1) no statistically significant difference was observed in the incidence of neurofibromas, Lisch nodules, and bone lesions between the groups with normal and altered VEP findings (Figure 3), while (2) a statistically significant more frequent occurrence of OPG (*p* = 0.008), tumors (*p* = 0.032), epilepsy (*p* = 0.043) and cognitive disorders (*p* = 0.028) was observed in the group of patients with a pathological finding of VEP (Figures 4, 5).

**Table 1:** Comparison of the frequency of clinical manifestations of NF type 1 observed in our study with the data from available literature (24–27).

	Our study	Available literature
Café-au-lait macules	100%	95%
Skin fold freckles	100%	90%
Lisch nodules	63%	92%
Neurofibromas	67%	50–90%
Optic pathway gliomas	33%	15–20%
Bone malformations	30%	10–50%
Tumours	50%	25%
Cognitive disorders	37%	30–60%
Epilepsy	15%	6–13%

**Figure 6:**
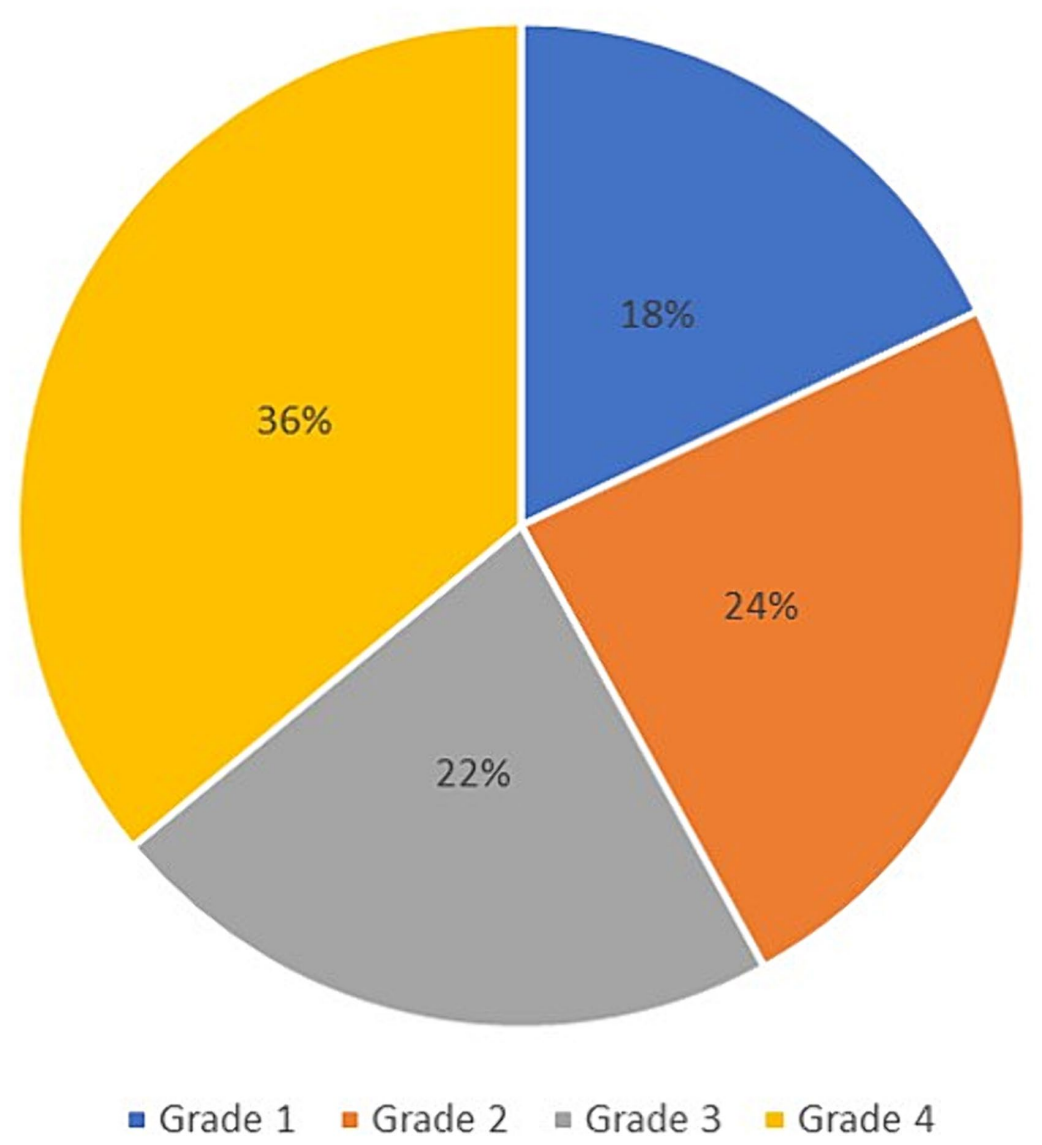
Distribution of patients from examined group according to grades of Riccardi scale.

**Figure 7:**
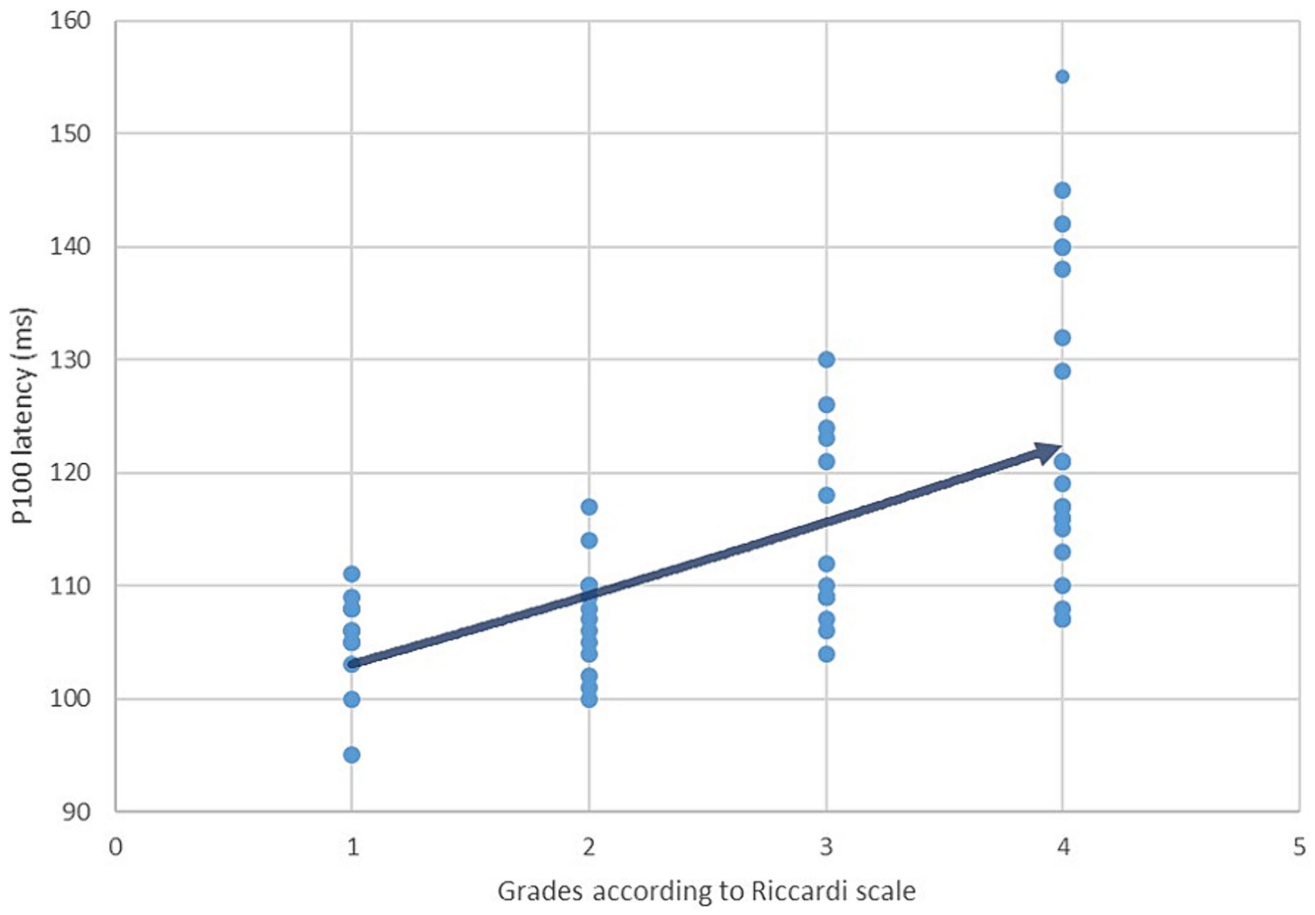
Correlation between P100 latency values and the severity of the clinical manifestations in patients with NF type 1 defined using Riccardi scale (Spearman coefficient *r*_s_ = 0,665).

The final data analysis level assessed the correlation between the prolongation of P100 latency and the severity of the clinical picture in patients with NF type 1, defined using the Ricardi scale with four grades. According to the mentioned scale, 18% of patients met the criteria for entering grade 1, 24% for grade 2, 22% for grade 3, and 36% of patients for grade 4 (Figure 6). Ultimately, the Spearman rank correlation test showed a moderately strong positive correlation (*r*_s_ = 0.665) between the prolongation of P100 latency and the severity of the clinical picture (Figure 7).

## 4 Discussion

One of the essential characteristics of neurofibromatosis type 1 is complete penetrance but highly variable expressivity, which means that the disease will manifest in all patients but with different severity in the clinical picture (28). Bearing all this in mind, one of the most critical questions that our study tried to answer is whether the PR-VEP finding can serve as a diagnostic parameter based on which we will be able to predict, in the condition of significant variations in expressivity, the behavior of the disease in each patient with NF type 1.

Initially, it is necessary to comment on the calculated threshold value for P100 latency, which in our study was 116 ms. Official textbooks and standards emphasize the necessity of calculating cut-off values for each population separately by summing the arithmetic mean values for P100 latency and 2.5 standard deviations (8, 22). The obtained P100 latency value of 116 ms agrees with the recommendations given by Drislane et al. (29), who advise that such a threshold value can be found in the reference range of 114 to 117 ms.

The frequencies of clinical manifestations of NF type 1 observed in our study are comparable to the greatest extent with the data found in the available literature (24–27). Compared to previous studies, neurofibromas and Lisch’s nodules occur less frequently in our study group, while OPG and benign and malignant tumors are more common. The observed differences in the prevalence of clinical manifestations can be explained in several ways. Firstly, the appearance of symptoms and signs of NF type 1 is age-dependent, which means that differences in the age of studied populations will contribute to significant variations in the frequency of specific clinical characteristics (30). Secondly, bearing in mind that one of the criteria for the inclusion in our study was a VEP analysis, the more frequent occurrence of OPG can be attributed to selection bias.

By analyzing the frequency distribution of clinical characteristics between the groups of patients with normal and altered VEP findings, we concluded that neurofibromas, Lisch’s nodules, and characteristic bone lesions (scoliosis, dysplasia of the sphenoid bone, and bowing of the long bones of the extremities) occur with equal frequency in both groups. On the other hand, a statistically significant higher frequency of OPG was shown in the group of patients with pathological VEP. This finding was expected because the literature has documented the prolongation of P100 latency and the reduction of P100 wave amplitude in people with this tumor (31, 32). However, it is important to highlight several facts related to the diagnostic value of VEP when it comes to OPG. In our study, the prolongation of P100 latency during binocular stimulation was used as a criterion for qualifying VEP findings as pathological, resulting in a relatively low sensitivity of VEP in diagnosing OPG (SN = 55%). Suppose the prolongation of P100 latency during monocular stimulation, reduction of P100 wave amplitude below 5 μV, and altered morphology or complete absence of P100 waves are included as additional criteria; In that case, the sensitivity of VEP in detecting OPG increases to 90–100% (14). Because of its extremely high sensitivity and better cost-effectiveness ratio, some authors have suggested that VEP be used as a screening method for OPG in patients with NF type 1 instead of magnetic resonance imaging (15). It shows also superior results when compared to ophthalmologic examination (visual acuity testing), especially in pediatric population because of the lack of compliance and cooperativity (33–35). Nevertheless, this issue is still a subject of debate, and VEP analysis is currently used in the follow-up of disease progression in patients with a diagnosis of OPG.

In addition to the increased prevalence of OPG, in the group of patients who had a pathological finding of VEP, a statistically significant, more frequent occurrence of benign and malignant tumors, as well as epilepsy and cognitive disorders, was also observed. The increased frequency of altered VEP findings in patients with cognitive disorders is the most exciting result we have reached in our study. Although VEP is conventionally used as a diagnostic tool that assesses the integrity of the visual pathway, some modern studies have concluded that the finding of VEP can reflect the overall activity pattern of the cerebral cortex and that, as such, it can be considered a helpful tool that detects various disorders of higher cortical functions (36). This should not surprise us if we consider that the main generator of P100 waves is the secondary visual cortex, in which visual stimuli are processed and integrated with impulses from other parts of the brain (8). This visual field is located in the broader band of the associative parieto-occipital cortex, which, together with the prefrontal cortex, provides the anatomical substrate for a whole range of executive functions, including attention, memory, working memory, inhibitory control, and visuospatial orientation (37). Disruption of these executive functions can be manifested clinically as a learning disorder in children of school age suffering from NF type 1. These learning problems can be classified as specific (dyslexia and dyscalculia) or mixed learning problems (7). Based on animal models with heterozygous *Nf*+/*Nf*− mutation, increased GABAergic transmission in inhibitory interneurons of the hippocampus and striatum was identified as the main culprit for the mentioned disorders (38). Namely, the activity of the ras/RAF/MEK/ERK signaling pathway is increased due to the loss of function of neurofibromin, which leads to the phosphorylation of synapsin and increased release of GABA from synaptic vesicles (39). The consequence of this neurotransmitter‘s increased secretion is a disturbance of the balance between inhibitory and excitatory impulses at the CA1 zone of the hippocampus, which leads to a decrease in long-term potentiation and synaptic plasticity. These two processes play a vital role in memory and learning (40).

Further evidence that VEP can be used to assess the extent of synaptic plasticity is provided in a study by Castricum et al. (19), which is based on the assumption that after the application of repetitive visual stimuli, there is a change in the response of the visual cortex to the repeated stimulus through an increase in the amplitude of the P100 wave, which is an electrophysiological correlate of the memory process (41). Their research showed reduced plasticity of visual evoked potentials in people with NF type 1. Knowledge of complex signaling pathways and their influence on the central neurotransmitter systems enabled the introduction of new pharmacotherapeutic approaches. It is encouraging that, at least on an experimental level, it has been demonstrated that the administration of lovastatin (an inhibitor of ras protein activation) and picrotoxin (a GABA-A channel antagonist) can lead to a significant improvement of the previously mentioned cognitive disorders (7).

At the very end, a moderately strong positive correlation between the prolongation of P100 latency and the severity of the clinical picture in patients with NF type 1 indicates the possibility of utilizing the pathological finding of VEP as a predictor of the appearance of severe disease manifestations. To the best of the authors’ knowledge, our study was among the first to examine the possibility of such a correlation. We can interpret the results we have reached in the first place as an attempt to characterize a unique clinical phenotype of NF type 1, the main characteristics of which are an altered VEP finding and more frequent occurrence of severe complications such as malignant tumors, pharmacoresistant epilepsy, and numerous others. Another important implication is the possibility of including patients with a pathological finding of VEP and a complex clinical picture in modern therapeutic protocols where the drug selumetinib (a MEK tyrosine kinase inhibitor) is used (42, 43). Although selumetinib is currently only recommended for patients with severe forms of plexiform neurofibromas, the future of research lies in the possibility of using this therapy for patients with a severe form of NF type 1. In this case, VEP analysis would be essential in selecting patients with a pathological finding that assumes the existence of benign and malignant tumors.

## 5 Conclusion

NF type 1 is one of the most common genetic diseases in humans, characterized by significant variability in disease manifestations and the severity of the clinical picture. Our research examined the role of VEP in the diagnostic workup of patients with NF type 1. The results we reached showed a statistically significant more frequent occurrence of GOP, benign and malignant tumors, cognitive disorders, and epilepsy in the group of patients who had an altered VEP finding. It was also shown that there is a moderately strong positive correlation between the prolongation of P100 latency and the severity of the clinical picture expressed through the Riccardi scale. Bearing all this in mind, the authors of this study propose to include VEP analysis as a reliable and straightforward method in the diagnostic algorithms that will be used not only for the assessment of OPG but also for neurocognitive disorders and more severe forms of NF type 1. Finally, the results indicate the necessity of additional research that will determine the role of VEP in characterizing the biological behavior of NF type 1 in each patient in a more precise manner.

## Data availability statement

The original contributions presented in the study are included in the article/supplementary material, further inquiries can be directed to the corresponding authors.

## Ethics statement

The studies involving humans were approved by Clinic of Neurology and Psychiatry for Children and Youth Ethics Committee. The studies were conducted in accordance with the local legislation and institutional requirements. Written informed consent for participation in this study was provided by the participants’ legal guardians/next of kin.

## Author contributions

JJ: Conceptualization, Formal analysis, Methodology, Validation, Writing – original draft, Writing – review & editing. NZ: Data curation, Investigation, Visualization, Writing – original draft. BN: Conceptualization, Methodology, Validation, Writing – original draft. NI: Data curation, Formal analysis, Software, Writing – review & editing. BR: Data curation, Formal analysis, Software, Writing – review & editing. DN: Supervision, Validation, Writing – review & editing.
